# A comprehensive tool for measuring mammographic density changes over time

**DOI:** 10.1007/s10549-018-4690-5

**Published:** 2018-02-01

**Authors:** Mikael Eriksson, Jingmei Li, Karin Leifland, Kamila Czene, Per Hall

**Affiliations:** 1 Department of Medical Epidemiology and Biostatistics, Karolinska Institutet, Nobels väg 12A, 171 77 Stockholm, Sweden; 2 Human Genetics, Genome Institute of Singapore, Singapore, Singapore; 3 Department of Surgery, National University of Singapore, Singapore, Singapore; 4 Department of Radiology, South General Hospital, Stockholm, Sweden; 5 Department of Oncology, South General Hospital, Stockholm, Sweden

**Keywords:** Mammographic density, Breast cancer, Risk, Recurrence, Endocrine treatment, Therapy response, Longitudinal, Time series

## Abstract

**Background:**

Mammographic density is a marker of breast cancer risk and diagnostics accuracy. Density change over time is a strong proxy for response to endocrine treatment and potentially a stronger predictor of breast cancer incidence. We developed STRATUS to analyse digital and analogue images and enable automated measurements of density changes over time.

**Method:**

Raw and processed images from the same mammogram were randomly sampled from 41,353 healthy women. Measurements from raw images (using FDA approved software iCAD) were used as templates for STRATUS to measure density on processed images through machine learning. A similar two-step design was used to train density measures in analogue images. Relative risks of breast cancer were estimated in three unique datasets. An alignment protocol was developed using images from 11,409 women to reduce non-biological variability in density change. The protocol was evaluated in 55,073 women having two regular mammography screens. Differences and variances in densities were compared before and after image alignment.

**Results:**

The average relative risk of breast cancer in the three datasets was 1.6 [95% confidence interval (CI) 1.3–1.8] per standard deviation of percent mammographic density. The discrimination was AUC 0.62 (CI 0.60–0.64). The type of image did not significantly influence the risk associations. Alignment decreased the non-biological variability in density change and re-estimated the yearly overall percent density decrease from 1.5 to 0.9%, *p* < 0.001.

**Conclusions:**

The quality of STRATUS density measures was not influenced by mammogram type. The alignment protocol reduced the non-biological variability between images over time. STRATUS has the potential to become a useful tool for epidemiological studies and clinical follow-up.

**Electronic supplementary material:**

The online version of this article (10.1007/s10549-018-4690-5) contains supplementary material, which is available to authorized users.

## Introduction

High mammographic density is a strong risk factor for breast cancer [1]. The density consists of epithelium and stroma and is radiographically dense. Epithelium and stroma appears bright on a mammogram while the fatty tissue is radiolucent and appears dark. Density decrease is also a good proxy for therapy response to endocrine therapy, both in the preventive [2] and adjuvant settings [3, 4]. However, mammograms from the same woman at different time-points are not always comparable since dissimilar proportions of the breast are sometimes captured in the images. A difference in density with non-biological meaning could therefore be captured. A possible solution is to align images to make the amount of breast tissue similar in each image. Cumulus is the gold standard to measure mammographic density on analogue mammograms [5]. The drawbacks of Cumulus are that it does not account for dissimilar breast proportions in the images; it is labour intense, and heavily dependent on the reader skill [6]. Several commercial tools measure density automated on digital raw mammograms [7]. Raw images are available during a short time in the hospital work-flow before they are converted to processed images. Most often only processed images are stored for future use. Vendors of mammography machines use different conversion methods which makes processed images from different machines difficult to compare. Tools are also developed for measuring density on processed images [8]. However, there is currently no automated tool that measures density of raw and processed images regardless of vendor and accounts for technical difference between images from the same women. This is unfortunate since most digital images are stored as processed images and precise measures are needed to monitor treatment response and density change over time.

We previously showed that it is possible to measure mammographic density fully automated on analogue film mammograms [9]. Here we present a new algorithm which measures density on all type of images, regardless of vendor, and controls for non-biological differences seen in time series of mammograms from the same women.

## Method

### Three Swedish datasets

The KARMA cohort includes 70,877 women who attended mammography screening between January 2011 to March 2013 at any of four mammography units in Sweden [10]. Participants donated blood, answered a web-based questionnaire, and raw and processed digital mammograms were stored. Women reported length and weight, family history of breast cancer, age at menarche, parity, age at first child, menopausal status, and ever use of hormone replacement therapy (HRT). Breast cancer cases, invasive and in situ, were identified through the Swedish Information Network for CAncer treatment (INCA) national quality register.

The population-based LIBRO1 study included invasive and in situ breast cancer cases diagnosed between 2001 and 2008 in the Stockholm area. Frequency matching was used to age-match 2443 breast cancer cases with the available controls from the KARMA study. The third Swedish study was the population-based SASBAC study which included 1194 women diagnosed with invasive and in situ breast cancer between 1993 and 1995, and 1086 controls density sampled and frequency matched on age [9]. Pre-diagnostic analogue films were collected for all cases, and images closest to recruitment date were collected for the controls. The cases and controls in LIBRO1 and SASBAC contributed with the same lifestyle factors as was collected in KARMA.

### Density measures

In all, 41,353 breast cancer-free women were sampled from KARMA with available digital raw and processed images from the same mammograms (vendors General Electric, Philips, Sectra, Hologic, Siemens, Array Corp.). Mammographic density was measured on the raw mammograms using the FDA approved density measurement tool iCAD (iReveal^®^, Nashua, NH, USA), which served as the reference measure for STRATUS. STRATUS analysed 1027 image features of the processed and raw images from the same mammogram (Supplementary Text 1, [11]). STRATUS further learned how to estimate density on the processed images using machine learning by relating the 1027 feature variables with the known original reference density measure from the raw image of the same mammogram (Supplementary Text 2, [12]). The accuracy of the measurements was tested in an independent validation dataset. This two-step procedure with training and validation was performed for each type of mammogram and mammography machine using up to 4000 mammograms per machine to generate the density measures.

Density measures for analogue images were developed with all available women in the SASBAC study [9]. The density measures were trained using the same algorithm as for digital images here by learning on one of the breasts and validating on the contralateral breast.

### Risk estimation and discrimination

Using samples based on augmentation sampling [13] from the described datasets, we estimated the association between the density measurements derived from different kind of images and breast cancer incidence. Cases and controls with different types of mammograms were contrasted. The first risk estimation was done on a nested case–control study sample with a two-year follow-up using the available 433 incident breast cancer cases age-matched in one-year bands with 1732 controls in KARMA (Table 1). The risk association was estimated using density measures of the raw and processed mammograms, respectively (contrasting raw cases to raw controls; processed cases to processed controls). The second risk estimation set was defined as the 2443 LIBRO1 cases age-matched in one-year bands with the available 2999 controls from KARMA (analogue cases to digital controls). The third risk estimation set was defined as the 1194 breast cancer cases in LIBRO1 possible to age-match in one-year bands with the available 1086 controls from SASBAC (analogue cases to analogue controls).

**Table 1:** Description of the three case–control study samples used to calculate risk of breast cancer of mammographic density measured by STRATUS

Characteristics by study sample	Cases	Controls
KARMA study sample		
Number of participants	433^a^	1732^a^
Age (years), mean (SD)	57.4 (9.2)	57.4 (9.2)
Ever use of HRT (%)	39	36
Postmenopausal (%)	65	65
Family history of breast cancer (%)	19	13
LIBRO1/KARMA study sample		
Number of participants	2443^b^	2999^a^
Age (years), mean (SD)	60.8 (9.5)	60.8 (9.5)
Ever use of HRT (%)	53	34
Postmenopausal (%)	92	90
Family history of breast cancer (%)	20	13
LIBRO1/SASBAC study sample		
Number of participants	1194^b^	1086^b^
Age (years), mean (SD)	63.1 (6.4)	63.1 (6.4)
Ever use of HRT (%)	54	50
Postmenopausal (%)	100	100
Family history of breast cancer (%)	15	8

Total women in the three study samples: 2876 cases and 5817 controls

^a^Digital images

^b^Analogue images

### Alignment of time series images

**Fig. 1:**
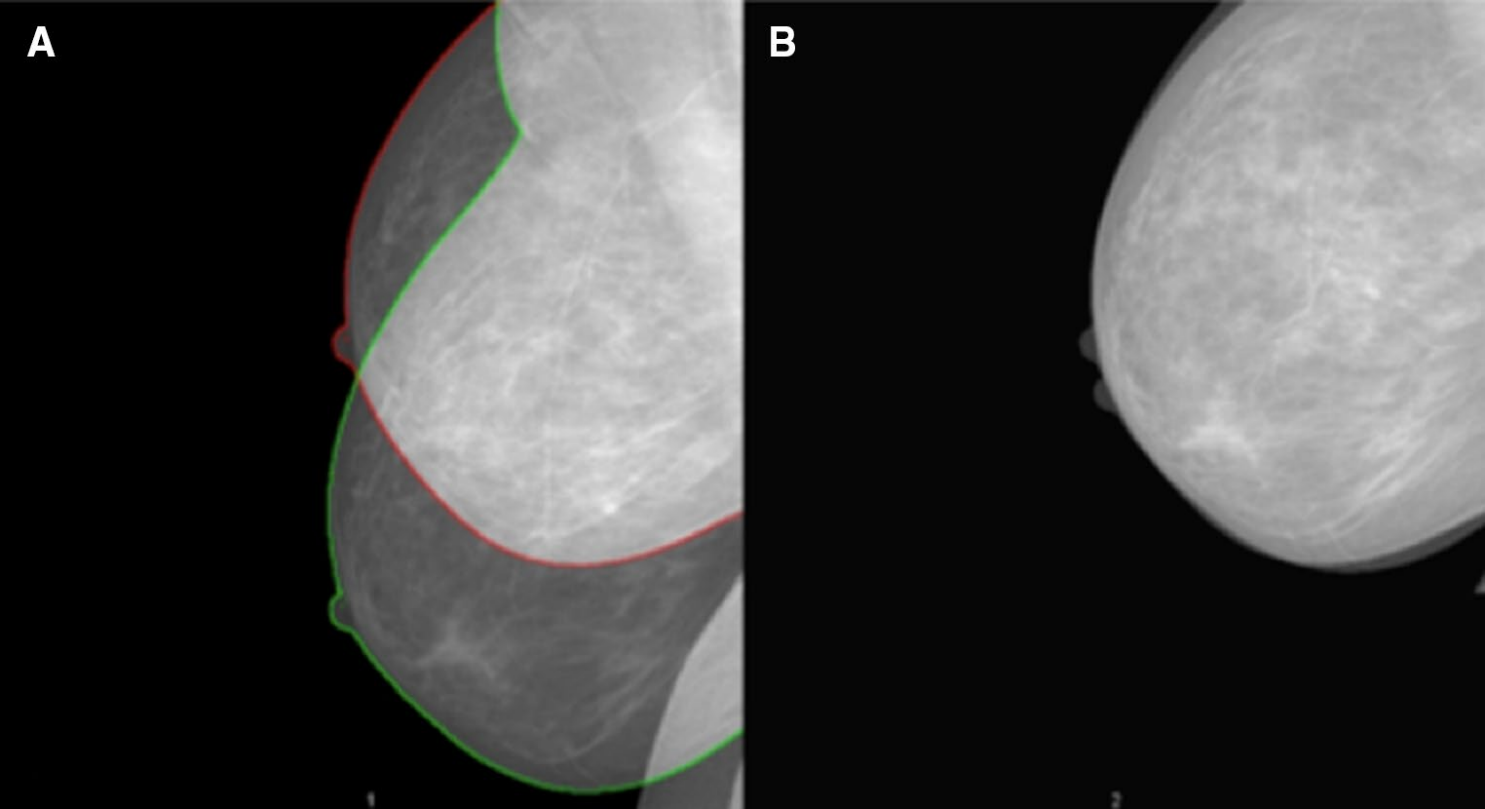
Two mammograms of the same breast were taken 2 min apart by the same radiographer. In Frame **a**, the mammograms were superimposed to show the difference in breast placement in the mammography machine. In Frame **b**, the two images were digitally aligned to the image showing the smallest breast size (outlined with red in Frame A) prior to density measurement

The problem with not aligning images becomes evident when looking at Fig. 1, Frame A. Two images from the same woman have been superimposed on each other. Most of the breast is seen in the image showing a green border. In contrast, the image with the red border lacks a large part of the breast and thereby also a part of the dense area. The red outlined breast area is 185 cm^2^. The corresponding area is 197 cm^2^ for the green outlined breast. In Fig. 1, Frame B the two images are aligned, and the two breast areas are now 185 cm^2^.

The tool for aligning images was created using the ImageJ program [14] and the TurboReg [15] plugin (Supplementary Text 3, [16, 17]).

Two datasets were used to evaluate the tool. For 11,409 KARMA participants, two mammograms were taken within minutes which gave the opportunity to study differences in density that possibly could not be due to biological alterations. The reasons for a second mammogram were not given in the medical records but we can assume that major reasons were technically suboptimal images and identification of artefacts. Second, we used all KARMA participants that had been through two rounds of negative screens (*N* = 55,073) to test if density measures of aligned images differed from the regular density measures. Dense area was measured in left or right breast at each screen and the average dense area was calculated.

### Statistical methods

The agreements between the STRATUS and reference density measures were investigated using Spearman’s rank correlation coefficient [18] and Bland–Altman fit plot [19].

The differences between the mammographic density measures of different mammogram types were investigated using least square means of mammographic percent density adjusted for age, BMI, and two mammography machine related factors, i.e. voltage of the X-ray tube and thickness of the compressed breast.

The association between mammographic density and breast cancer was estimated using conditional logistic regression in the three case–control study samples separately and in all study samples combined. Three models were constructed to assess potential confounders for the risk association. The first model included percent density and age, the second model also included BMI, and the full model also ever use of HRT, menopause status, and family history of breast cancer. The addition of X-ray tube voltage, breast thickness, and indicators of mammogram type and study sample to the full model did not change the estimates and were therefore excluded in the final model.

The odds ratios were calculated per standard deviation with 95% Wald confidence intervals. The discrimination performances of the models were calculated with area under the receiver operating curve (AUC) and 95% Wald confidence intervals.

The effect of image alignment was calculated by first subtracting the last measure from the first for aligned and non-aligned images, respectively. The density differences were further aggregated as means and standard deviations for the aligned and non-aligned images. Levene’s test [20] and the Student’s *t* test [21] were used to test for differences in standard deviations and means between the aligned and non-aligned image measures.

The analysis of the longitudinal density measures was performed by first calculating the density change per year for aligned and non-aligned images, respectively. The change in dense area per year was calculated by subtracting the last measure from the first and divide by the number of years between examinations. The differences between means and standard deviations of density changes in aligned and non-aligned images were calculated similarly using the Student’s *t* test and the Levene’s test. The density change per year was stratified by age and BMI and modelled using non-linear regression.

All tests were two-sided with 5% significance level. All analyses were performed using the statistical software SAS v9.4.

## Results

In total, 45,417 women from the KARMA, LIBRO1, and SASBAC studies contributed with raw and processed mammograms from nine different types of mammograms from six vendors (Supplementary Table 1). The correlations between the measures on the raw and processed mammogram were close to 0.9 (Supplementary Fig. 1). The correlations increased with increasing number of images used in the density training session and reached Spearman *r* = 0.933 (min = 0.923, max = 0.936) with 4000 images per machine. The Bland–Altman fit plot showed agreement between the raw and processed mammograms and the standardized mean difference was 0.01 with standard deviation 0.28 (Supplementary Fig. 2). No significant differences were found in mean percent mammographic densities between the nine mammogram types after adjusting for age, BMI, X-ray tube voltage, and breast thickness, *p* > 0.05 (Supplementary Fig. 3). The same non-significant differences between mammography machines were seen when BMI was substituted with breast area as adjustment factor (data not shown).

The density risk association was estimated in three case–control study samples (Table 1). The odds ratios for percent density in the full model ranged between 1.5 (CI 1.3–1.7) and 1.7 (CI 1.6–1.8) per standard deviation, and the combined odds ratio was OR 1.6 (1.3–1.8) (Table 2).

**Table 2:** Odds ratios and 95% confidence intervals of breast cancer in three unique case–control study samples contrasting the performance of estimates per standard deviation from density measures in processed, raw, and analogue mammograms

Case–control study sample	Model 1^e^	Model 2^f^	Model 3^g^
KARMA (processed)^a^	1.6 (1.5–1.7)	1.7 (1.6–1.8)	1.7 (1.6–1.8)
KARMA (raw)^b^	1.6 (1.5–1.7)	1.7 (1.6–1.8)	1.7 (1.6–1.8)
LIBRO1/KARMA (processed/analogue)^c^	1.5 (1.4–1.6)	1.6 (1.4–1.8)	1.6 (1.4–1.8)
LIBRO1/SASBAC (analogue)^d^	1.5 (1.3–1.7)	1.5 (1.3–1.8)	1.5 (1.3–1.7)
Study samples combined	1.5 (1.3–1.6)	1.6 (1.3–1.8)	1.6 (1.3–1.8)

^a^433 cases and 1732 controls with density measurements from processed mammograms

^b^433 cases and 1732 controls with density measurements from raw mammograms

^c^2443 cases with density measurement from processed mammograms and 2999 controls with density measurements from analogue mammograms

^d^1194 cases and 1086 controls with density measurement from analogue mammograms

^e^Model 1—percent density and age

^f^Model 2—percent density, age, and BMI

^g^Model 3—percent density, age, BMI, ever use of HRT, menopause status, and family history of breast cancer

The discrimination performance of the full model ranged between AUC 0.60 (CI 0.57–0.63) and 0.63 (CI 0.60–0.65) in the three study samples; and the combined study sample AUC was 0.62 (0.60–0.64) (Table 3).

**Table 3:** Discrimination performance (AUC) and 95% confidence intervals in three unique case–control study samples contrasting the performance of estimates from density measures in processed, raw, and analogue mammograms

Case–control study sample	Model 1^e^	Model 2^f^	Model 3^g^
KARMA (processed)^a^	0.59 (0.55–0.63)	0.62 (0.59–0.65)	0.63 (0.60–0.65)
KARMA (raw)^b^	0.59 (0.55–0.63)	0.62 (0.59–0.65)	0.63 (0.60–0.65)
LIBRO1/KARMA (processed/analogue)^c^	0.59 (0.55–0.62)	0.60 (0.57–0.64)	0.62 (0.59–0.64)
LIBRO1/SASBAC (analogue)^d^	0.58 (0.55–0.62)	0.59 (0.55–0.63)	0.60 (0.57–0.63)
Study samples combined	0.59 (0.55–0.62)	0.60 (0.59–0.63)	0.62 (0.60–0.64)

^a^433 cases and 1732 controls with density measurements from processed mammograms

^b^433 cases and 1732 controls with density measurements from raw mammograms

^c^2443 cases with density measurement from processed mammograms and 2999 controls with density measurements from analogue mammograms

^d^1194 cases and 1086 controls with density measurement from analogue mammograms

^e^Model 1—percent density and age

^f^Model 2—percent density, age, and BMI

^g^Model 3—percent density, age, BMI, ever use of HRT, menopause status, and family history of breast cancer

**Fig. 2:**
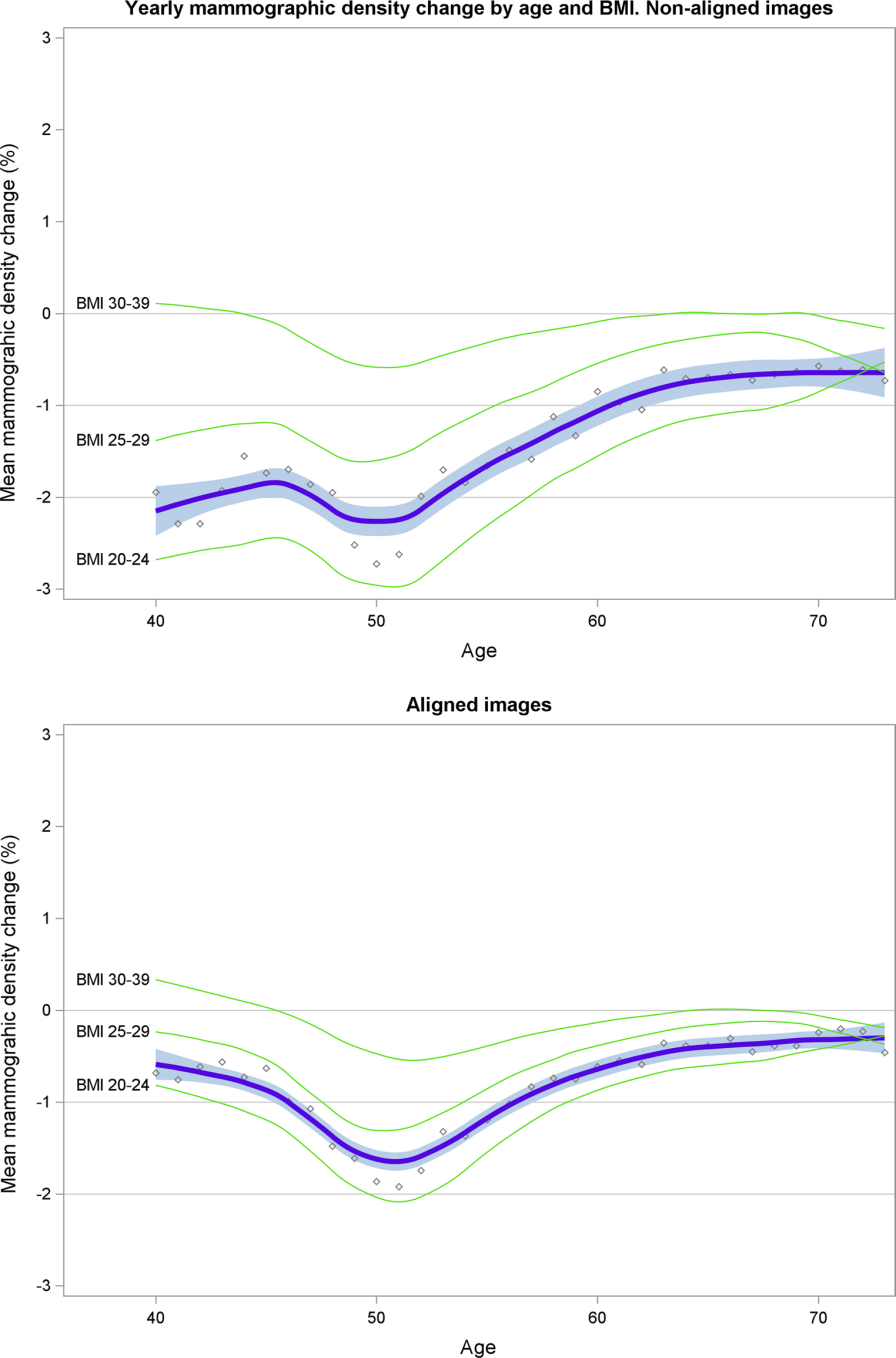
Comparison of yearly percent mammographic density change in 55,073 women with aligned and non-aligned mammograms taken at two time-points 1–2 years apart. The blue fitted curve (non-linear regression) shows the yearly average percent density change with 95% CI. The circled dots show the density averages by age at baseline. The green curves show the density change stratified by BMI at baseline for women with BMI between 20 and 40

The aligned percent density measures showed significantly lower variability compared to the non-aligned percent density measures (SD 8.0 vs. 28.6, *p* < 0.001) in the 11,409 women who had two consecutive mammograms taken within minutes (Table 4). The aligned percent density measures also showed significantly lower yearly decrease compared to the non-aligned density measures for the 55,073 women who had mammograms taken 1–2 years apart, 0.9 versus 1.5 (SD 4.3 vs. 5.0, *p* < 0.001), Table 4. In Fig. 2 the yearly, non-aligned (upper panel) and aligned (lower panel), percent density changes were plotted for the 55,073 women. The blue fitted lines show the yearly average percent density change with 95% CI by age at baseline. The green curves show the density change stratified by BMI subgroups defined at baseline. The biggest difference between aligned and non-aligned measures is seen during women’s fertile part of life. The yearly mean percent density decreases in 40-year-old women (*N* = 2499) was 1.9 (95% CI 1.7–2.2) using non-aligned images and 0.7 (95% CI 0.4–0.9) using aligned images.

**Table 4:** Comparison of variability in density measurements of non-aligned and aligned mammograms taken at two time-points

Time series	Non-aligned	Aligned	*p* value^c^
Mammograms within minutes apart (last minus first)^a^			
Difference in percent density, mean (SD)	0.3 (20.6)	0.1 (10.4)	< 0.001
Difference in dense area cm^2^, mean (SD)	0.3 (28.6)	0.0 (8.0)	< 0.001
Mammograms up to 2 years apart (last minus first)^b^			
Yearly change in percent density, mean (SD)	− 1.5 (5.0)	− 0.9 (4.3)	< 0.001
Yearly change in dense area cm^2^, mean (SD)	− 1.8 (6.8)	− 1.8 (6.8)	0.99

^a^*N* = 11,409 women with digital images. Images taken on average 1 min apart. Mean age 57 (SD 9,8), BMI 26 (SD 4.7)

^b^*N* = 55,073 women with digital processed images. Images taken on average 2.0 years apart. Mean age 55 (SD 10.0), BMI 25 (SD 4.2)

^c^The Levene’s test tested for equality of variances between the measures of aligned and non-aligned mammograms. All density measurement differences were normally distributed

The Spearman rank coefficient r was used to calculate the correlation between the density measurements of aligned and non-aligned mammograms. The correlation between measures from aligned and non-aligned mammograms taken up to 2 years apart was *r* = 0.64 for percent density and *r* = 0.60 for dense area

## Discussion

We created a tool that enables comparison of mammographic density changes over time without being restricted to type of mammogram or technical differences between images. STRATUS performs high-throughput measurements of mammographic density on mammograms from different mammography machines and mammogram types. As a consequence, risk assessments were not influenced by type of image when estimated in three independent study samples which included a combination of different mammogram types. The alignment protocol also reduced the non-biological variability between mammograms.

Mammographic density is a strong marker of breast cancer risk with a discrimination performance comparable to established risk models, which combine information on hormonal exposures and family history of breast cancer [22].

There are several reasons for identifying the true density change over time. A longitudinal study showed that individual differences in mammographic density changes over time were not associated with breast cancer risk [23]. This result was, however, based on non-aligned images. As revealed in Fig. 1, technical differences between mammograms influence the comparison of density measurements over time. Figure 2 shows that aligned density measurements capture the level and rate of density change different in comparison to non-aligned density measures. This is particularly true for premenopausal women and this could be a reflection of the change in breast size [24], percent density [25], and dense area [26] during the menstrual cycle [27]. Breast size is further strongly modified by BMI, which means that density measures are influenced also by changing BMI over time.

Another reason to study density change over time is that density change is a remarkably good proxy for treatment response in the adjuvant and preventive setting [28]. Studies have shown that a decrease in mammographic density by 20% during the first two years of adjuvant therapy reduced breast cancer mortality by nearly 50% over the subsequent 15 years [3, 4]. Patients that benefits from adjuvant anti-hormonal therapy could therefore be identified. Non-responders could also be identified early in the treatment and therefore be selected to alternative treatments. Third, breast cancer prevention has been demonstrated to reduce breast cancer incidence by approximately 50% after tamoxifen treatment [2]. A decrease in mammographic density in the first year is also here an excellent proxy for a decrease in breast cancer incidence in the following years. However, tamoxifen treatment is associated with side-effects, similar to menopausal symptoms, and in rare cases endometrial cancer and thromboembolism [28]. It is therefore crucial to treat only the group of healthy women that respond to therapy and are likely to benefit with a decrease in breast cancer incidence.

Several techniques are available for aligning images [29]. We tested translation, rigid body, scaled rotation, affine, and bilinear transformation. The translation registration method was not sensitive to breast tissue overlaying and preserved the largest part of the original breast area, and was used in the final analysis (Supplementary Text 3). The alignment protocol was developed to analyse several mammograms in a time series and to not be sensitive to differences in pixel intensities between processed and raw or analogue images. The alignment technique could potentially also be used for Cumulus in a post-processing step, if the reader saved the breast area and dense area positioning in the mammograms during the measurement procedure.

The strength of our study is that we used a large population-based cohort with access to both raw and processed images form the same examinations. We also had access to repeated and longitudinal measurements from the same women. In addition, we could construct case–control study samples and combine cases and controls with different type of images from three unique Swedish studies.

There should, however, be some caution interpreting our findings. Sweden has a well-organised screening program with mammograms of high quality. Further studies are needed to show how our risk estimates are affected by imaging techniques and radiographer routines in other countries. The time from date of mammogram to date of breast cancer diagnosis varied between the studies. The average time in the KARMA sample was 1.7 and 0.2 years in the other two study samples. However, the contralateral breast was used for the risk association analyses and the time differences seen in this study is not likely to affect the results [30]. The proportion of HRT users differed between the studies. HRT is positively associated with higher levels of density and may have affected the risk estimates. However, HRT was used as an adjustment factor in this study and did not seem to affect the risk estimates.

The density algorithm for digital mammograms was constructed solely on women with no diagnosis of breast cancer, while the density algorithm for analogue mammograms was developed on an equal number of healthy women and breast cancer cases. Although no significant difference was seen in the density measures from the two image types, the analogue density measures could be more susceptible to capture radio dense tissue associated with breast cancer compared to the density measures of digital mammograms.

## Conclusion

STRATUS is a fully automated tool that measures mammographic density from mammograms obtained from a variety of sources (raw and processed digital images, analogue films). The added alignment feature provided by STRATUS improves longitudinal measurements of mammographic density. Given that an increasing number of mammograms are stored in the screening and clinical setting, STRATUS-derived mammographic density can become a useful tool for risk prediction and treatment response in research and clinical praxis.

## Electronic supplementary material

Below is the link to the electronic supplementary material.

Supplementary material 1 (DOCX 23 kb)

Supplementary material 2 (DOCX 105 kb)

Supplementary material 3 (DOCX 12 kb)

## References

[1] Boyd NF, Byng JW, Jong RA, Fishell EK, Little LE, Miller AB, Lockwood GA, Tritchler DL, Yaffe MJ (1995). Quantitative classification of mammographic densities and breast cancer risk: results from the Canadian National Breast Screening Study. J Natl Cancer Inst.

[2] Cuzick J, Warwick J, Pinney E, Duffy SW, Cawthorn S, Howell A, Forbes JF, Warren RM (2011). Tamoxifen-induced reduction in mammographic density and breast cancer risk reduction: a nested case-control study. J Natl Cancer Inst..

[3] Li J, Humphreys K, Eriksson L, Edgren G, Czene K, Hall P (2013). Mammographic density reduction is a prognostic marker of response to adjuvant tamoxifen therapy in postmenopausal patients with breast cancer. J Clin Oncol.

[4] Nyante SJ, Sherman ME, Pfeiffer RM, Berrington de Gonzalez A, Brinton LA, Aiello Bowles EJ, Hoover RN, Glass A, Gierach GL (2015). Prognostic significance of mammographic density change after initiation of tamoxifen for ER-positive breast cancer. J Natl Cancer Inst.

[5] Byng JW, Yaffe MJ, Jong RA, Shumak RS, Lockwood GA, Tritchler DL (1998). Analysis of mammographic density and breast cancer risk from digitized mammograms. Radiographics.

[6] Burton A, Byrnes G, Stone J (2016). Mammographic density assessed on paired raw and processed digital images and on paired screen-film and digital images across three mammography systems. Breast Cancer Res.

[7] Chen JH, Gulsen G, Su MY (2015). Imaging breast density: established and emerging modalities. Transl Oncol.

[8] Stamatia D (2017). Qualitative versus quantitative mammographic breast density assessment: applications for the US and abroad. Diagnostics.

[9] Li J (2012). High-throughput mammographic density measurement: a tool for risk prediction of breast cancer. Breast Cancer Res.

[10] Gabrielsson M (2017). Cohort profile: the Karolinska mammography project for risk prediction of Breast Cancer (KARMA). Int J Epidemiol.

[11] http://dicom.nema.org/. Accessed 30 March, 2017

[12] Solomon SR, Sawilowsky SS (2009). Impact of rank-based normalizing transformations on the accuracy of test scores. J Modern Appl Stat Methods.

[13] Li (2015). Logistic analysis of epidemiologic studies with augmentation sampling involving re-stratification and population expansion. Biostatistics.

[14] National Cancer Institute, http://imagej.nih.gov/ij/. Accessed 15 March 2017

[15] http://bigwww.epfl.ch/thevenaz/turboreg/. Accessed 30 March 2017

[16] Levenberg Kenneth (1944). A method for the solution of certain non-linear problems in least squares. Q Appl Math.

[17] Marquardt Donald (1963). An algorithm for least-squares estimation of nonlinear parameters. SIAM J Appl Math.

[18] Spearman C (1907). Demonstration of formulæ for true measurement of correlation. Am J Psychol.

[19] Bland JM, Altman DG (1999). Measuring agreement in method comparison studies. Stat Methods Med Res.

[20] Levene H, Olkin I (1960). Robust tests for equality of variances. Contributions to probability and statistics; essays in honor of Harold hotelling.

[21] Student [William Sealy Gosset] (1908). The probable error of a mean. Biometrika 6:1

[22] Eriksson M (2017). A clinical model for identifying the short-term risk of breast cancer. Breast Cancer Res.

[23] Lokate M, Stellato RK (2013). Age-related changes in mammographic density and breast cancer risk. Am J Epidemiol Adv.

[24] Hussain Z, Roberts N (1999). Estimation of breast volume and its variation during the menstrual cycle using MRI and stereology. Br J Radiol.

[25] Chan S, Su MY (2011). Menstrual cycle-related fluctuations in breast density measured by using three-dimensional MR imaging. Radiology.

[26] Iversen A, Frydenberg H (2016). Cyclic endogenous estrogen and progesterone vary by mammographic density phenotypes in premenopausal women. Eur J Cancer Prev.

[27] Hovhannisyan G, Chow L (2009). Differences in measured mammographic density in the menstrual cycle. Cancer Epidemiol Biomark Prev.

[28] Mallick S, Benson R, Julka PK (2016). Breast cancer prevention with anti-estrogens: review of the current evidence and future directions. Breast Cancer..

[29] Thévenaz P, Ruttimann UE, Unser M (1998). A pyramid approach to subpixel registration based on intensity. IEEE Trans Image Process.

[30] Krishnan K, Baglietto L (2017). Longitudinal study of mammographic density measures that predict breast cancer risk. Cancer Epidemiol Biomark Prev.

