# Supplementary material for: A comprehensive tool for measuring mammographic density changes over time
Source: Breast Cancer Res Treat. 2018 Feb 1;169(2):371–9. doi: 10.1007/s10549-018-4690-5 (PMC5945741; doi:10.1007/s10549-018-4690-5)
Supplement: Supplementary file 1 — Supplementary material 1 (DOCX 23 kb) [file 10549_2018_4690_MOESM1_ESM.docx]

**Supplementary Text 1**

The Java based ImageJ programming framework developed by National Institutes of Health [ ^[[1]](#endnote-2)^ ], was used to develop STRATUS. STRATUS starts by reading the DICOM [^[[2]](#endnote-3)^] formatted full-field digital and analogue mammogram and for the digital images also the mammography machine acquisition parameters. STRATUS thereafter quality checks the mammograms by inspecting image size, availability of patient identifier, mammogram date and time, mammogram view position, description of the mammogram series and study, and breast implants. Dubious mammograms are marked in the output files. Images are further normalized to 200-micron pixel size.

Analog mammograms are cropped to remove the framing around the actual mammogram area. STRATUS flips the images, so the breast chest wall always appears to the left-hand side in the mammogram, and inverts the Look-Up-Table if the mammogram appears as a negative (i.e. when the pixel intensity representation is reversed).

STRATUS marks the breast area using threshold methods Intermodes, Triangle, or Means depending on the quality of the image. The next step identifies textures of the image in a cycle of 15 threshold methods (Otsu, RenyiEntropy, Huang, Intermodes, IsoData, Li, MaxEntropy, Mean, MinError, Minimum, Moments, Percentile, Shanbhag, Triangle, Yen) and one edge tracing method (Skeletonize). Twenty features (area, min, mean, max, std, modal, centroid, center, perimeter, bounding, fit, shape, integrated, median, skewness, kurtosis, limit, round, solidity, area_fraction) are measured for each cycle on the whole segmented breast area and also stratified by the size of textures in the breast area. Mammography machine acquisition parameters are added as extra information to the measures of image features including the x-ray exposure used (kilo volt and tube current) and the compression force and the thickness of the breast during compression. All features are measured for each threshold method and compiled into one row with up to 1,027 variables per digital mammogram. The variables are ordered starting with a variable holding the value for the first threshold method and first feature, i.e. OtsuArea. The second variable is OtsuMin and the last threshold-feature variable is YenSkeletonize.

STRATUS uses the same algorithm for analogue and digital images.

**Supplementary Text 2**

The learning step used the R programming framework developed by r-project.org and runs as a program within the framework. The STRATUS image analysis variables are loaded as an R dataset. The (raw) image reference density measures are matched to the corresponding processed image features generated by STRATUS. Scaled principal component analysis (PCA) is performed on the variables and a prediction dataset is created based on the PCA data. The original image reference measures (percent density, dense area, breast area) are transformed to a distribution close to normal form using square root transform [ ^[[3]](#endnote-4)^ ]. The R package “penalized” is used to fit a generalized linear penalized lasso regression model. One hundred loops of model fits with ten-folded cross-validation are used to calculate the mean lambda used to penalize the estimates for the final model. This procedure is done for percent density, dense area, and breast area. The outcome measures are then back transformed to original density distribution by powering the values with two.

**Supplementary Text 3**

The alignment tool uses the TurboReg plugin in ImageJ to read pairs of Full Field Digital Mammograms and to align the images. First, each breast area is marked using the threshold methods Intermodes, Triangle, or Means depending on the quality of the image. The binary masks from the breast area markings are used to guide the superimposition of the breast areas on top of each other in layers. The breast area masks are moved towards each other to the optimal position where their pixel intensities show minimal difference in least square means. The positioning technique is therefore not sensitive to aligning images with differences in pixel intensities such as seen in raw, processed or analogue mammograms. The actual breast area pixel information in the image is linked to the mask position and moved accordingly. The internal positioning of the pixels in the images is preserved (i.e. not distorted) during the move. This translation technique is based on the Marquardt-Levenberg algorithm [^[[4]](#endnote-5)^,^[[5]](#endnote-6)^]. Any parts of the images outside the mutual image information are cropped. Snap shots of the superimposed images before and after alignment are saved together with the moving coordinates.

1. <http://rsb.info.nih.gov/ij/index.html>. Accessed 30 March, 2017. [↑](#endnote-ref-2)
2. <http://dicom.nema.org/>. Accessed 30 March, 2017. [↑](#endnote-ref-3)
3. Solomon, S., R., & Sawilowsky, S. S. (2009). Impact of rank-based normalizing transformations on the accuracy of test scores. Journal of Modern Applied Statistical Methods, 8(2), 448 – 462. [↑](#endnote-ref-4)
4. [Levenberg, Kenneth](https://en.wikipedia.org/wiki/Kenneth_Levenberg) (1944). "A Method for the Solution of Certain Non-Linear Problems in Least Squares". *Quarterly of Applied Mathematics*. **2**: 164–168. [↑](#endnote-ref-5)
5. [Marquardt, Donald](https://en.wikipedia.org/wiki/Donald_Marquardt) (1963). "An Algorithm for Least-Squares Estimation of Nonlinear Parameters". *SIAM Journal on Applied Mathematics*. **11** (2): 431–441. [doi](https://en.wikipedia.org/wiki/Digital_object_identifier):[10.1137/0111030](https://dx.doi.org/10.1137%2F0111030) [↑](#endnote-ref-6)
