# Supplementary material for: A comprehensive tool for measuring mammographic density changes over time
Source: Breast Cancer Res Treat. 2018 Feb 1;169(2):371–9. doi: 10.1007/s10549-018-4690-5 (PMC5945741; doi:10.1007/s10549-018-4690-5)
Supplement: Supplementary file 2 — Supplementary material 2 (DOCX 105 kb) [file 10549_2018_4690_MOESM2_ESM.docx]

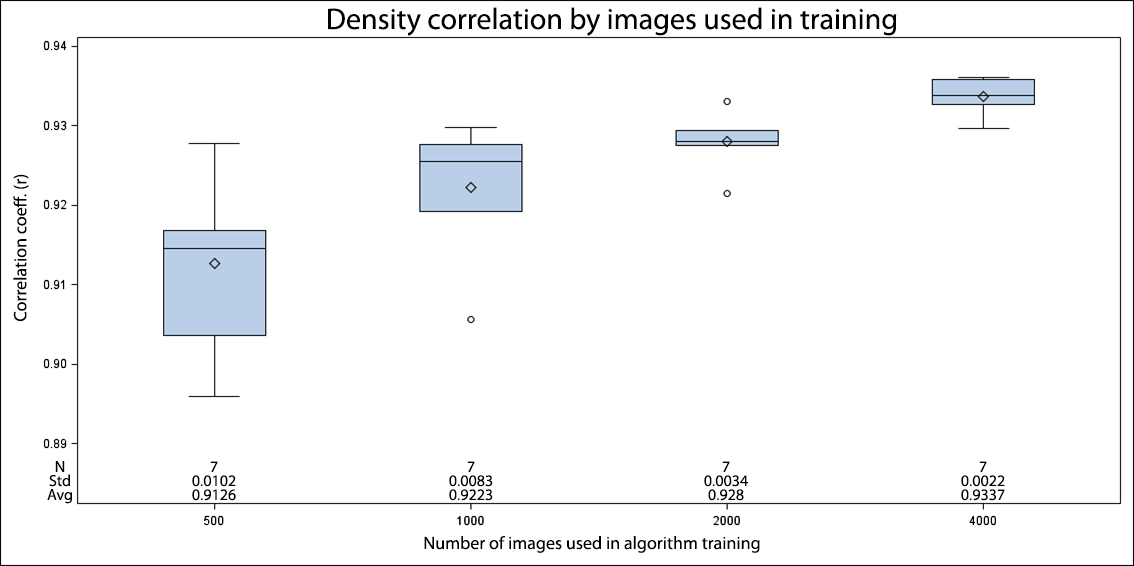


**Supplementary Figure 1.** Spearman rank correlation r of percent density between raw and processed images per type of mammography machine in relation to the number of mammogram pairs used in the training dataset for machine learning of STRATUS. The figure shows results from the validation dataset. Two mammography machines with less than 4,000 mammograms were excluded in this comparison (Siemens and Hologic).


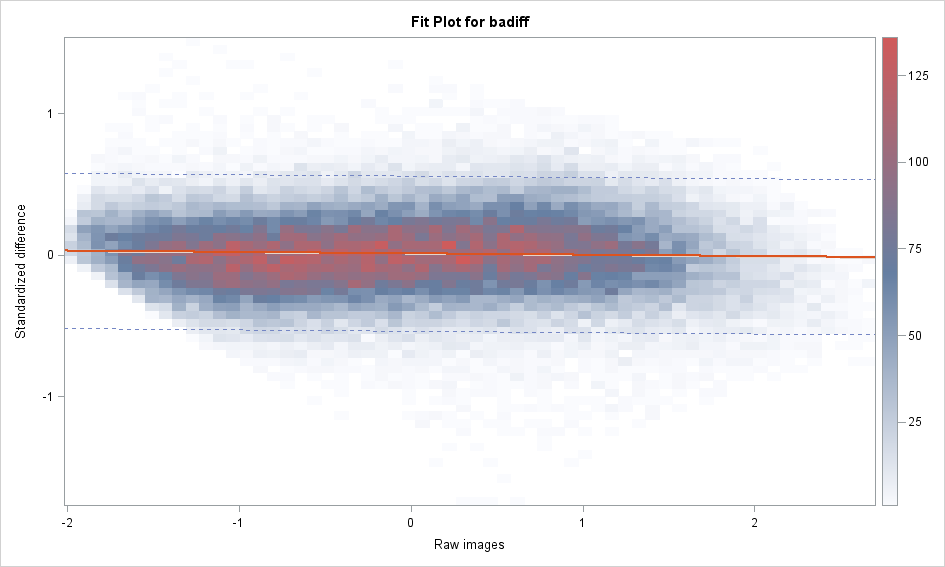


**Percent density agreement between raw and processed images**

**Supplementary Figure 2.** Bland-Altman fit plot with standardized percent density comparing the agreement with 95% CI (dotted lines) between raw and processed images in the validation dataset. The validation dataset included mammograms from all mammography machines trained with up to 4,000 mammograms. The graph was enhanced with red-to-white colour transition to indicate the number of density measures in the plot (right-hand scale). The standardized mean density difference between the raw and processed mammograms was 0.01 with standard deviation 0.28.


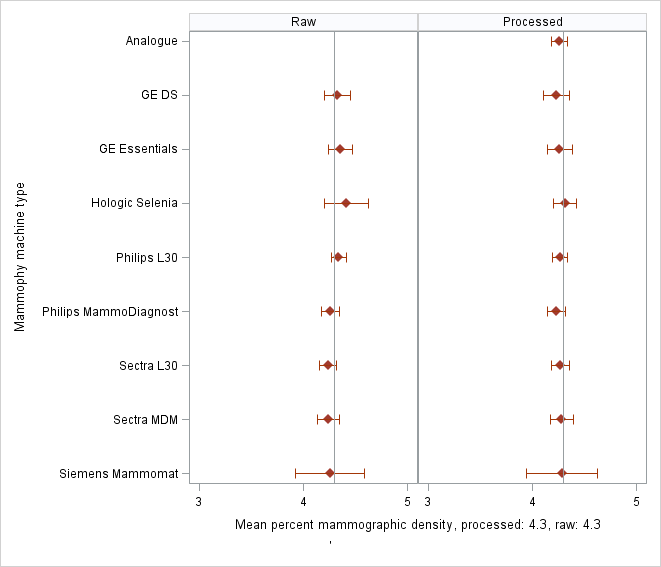


**Supplementary Figure 3.** Comparison of STRATUS mean percent mammographic densities and 95% confidence intervals between eight types of mammography machines with raw and processed mammograms; and the analogue mammogram type. Percent mammographic density was square root transformed (to a 0-10 scale) and adjusted for age, BMI, x-ray tube voltage, and the breast thickness recorded during compression in the machine. 39,186 women with processed images (also counting analogue images) were included as were 31,075 women with raw images. 28,908 women had both processed and raw mammograms. The number of mammograms was limited for the Siemens machine (N=136 processed and N=113 raw) and the Hologic machine (N=1,098 processed and N=214 raw) due to missing information on BMI for the women.

In a sub analysis, BMI was substituted with breast area as adjustment factor. This showed the same mean percent mammographic density of 4.3 in raw and processed mammograms with non-significant differences between the machines (data not shown).
