# Supplementary material for: A comprehensive tool for measuring mammographic density changes over time
Source: Breast Cancer Res Treat. 2018 Feb 1;169(2):371–9. doi: 10.1007/s10549-018-4690-5 (PMC5945741; doi:10.1007/s10549-018-4690-5)
Supplement: Supplementary file 3 — Supplementary material 3 (DOCX 12 kb) [file 10549_2018_4690_MOESM3_ESM.docx]

**Supplementary Table 1.** Number of women with processed and raw mammograms used in the development and validation of the STRATUS mammographic density measures.

| **Number of women per mammography machine type** | **Processed** | **Raw** |
| --- | --- | --- |
| Total number of women | 39,186 | 31,075 |
| Total number of women with raw and processed mammograms | 28,908 | 28,908 |
| GE Senograph Essentials | 8,043 | 9,069 |
| GE Senograph DS | 3,011 | 3,005 |
| Philips L30 | 6,152 | 6,136 |
| Philips Mammodiagnost DR | 1,443 | 1,355 |
| Sectra L30 | 5,999 | 4,974 |
| Sectra MDM (D40) | 5,422 | 6,209 |
| Hologic Selenia | 4,012 | 4,022 |
| Siemens Mammomat | 1,007 | 1,013 |
| Analogue (Array corp.) | 4,064 |  |
|  |  |  |

In total 41,353 KARMA women with processed or raw mammogram were included. Additional 4,064 women with analogue images were included. In total 45,417 women.

The analogue images were digitized using the Array 2905HD Laser Film Digitizer, which covers a range of 0 to 4.7 optical densities and a density resolution set to 12-bit dynamic range.
